# Development and validation of machine learning-based models integrating Septin9 methylation and serum biomarkers for early detection and differentiation of colorectal cancer

**DOI:** 10.7717/peerj.21053

**Published:** 2026-03-31

**Authors:** Cen Jiang, Yiyi Lu, Beiying Wu, Yunzhe Wu, Lilan Jin, Gang Cai, Zirui He, Lin Lin

**Affiliations:** 1 Department of Laboratory Medicine, Ruijin Hospital, Shanghai Jiaotong University School of Medicine, Shanghai, China; 2 Department of General Surgery, Ruijin Hospital, Shanghai Jiaotong University School of Medicine, Shanghai, China

**Keywords:** Colorectal cancer, Machine learning, Septin9, Methylation, Adenomas

## Abstract

**Background:**

Accurate risk stratification and early detection of colorectal cancer (CRC) are critical for improving patient outcomes and optimizing the use of colonoscopy; however, the diagnostic performance of existing biomarkers remains suboptimal. This study aimed to develop and evaluate machine learning (ML)-based models to facilitate individualized risk assessment and clinical decision-making for colorectal lesions.

**Methods:**

A total of 1,714 participants who underwent colonoscopy at Department of Gastrointestinal Surgery, Ruijin Hospital, Shanghai Jiaotong University School of Medicine were included. Participants were categorized into normal colonoscopy controls (*n* = 677) and high-risk colorectal diseases group (*n* = 1,037), with the latter further subdivided into adenomas (*n* = 376) and CRC (*n* = 661) subsets. Demographic characteristics and relevant laboratory data were collected. Variables significantly associated with high-risk colorectal conditions or CRC were identified using univariable and multivariable logistic regression analyses and incorporated into two independent nomogram-based ML models. Model performance was evaluated using the area under the receiver operating characteristic curve (AUC), calibration curve, and decision curve analysis (DCA). SHapley Additive exPlanations (SHAP) analysis was performed to determine each feature’s contribution.

**Results:**

Gender, age, hemoglobin (Hb), C-reactive protein (CRP), carcinoembryonic antigen (CEA), and Septin9 methylation were independent predictors of high-risk colorectal diseases, with the latter five also specific for CRC (*p* < 0.001). Two ML models were developed: one predicting the probability of high-risk colorectal diseases and the other distinguishing CRC from adenoma. Both models demonstrated strong discriminative ability with high AUCs and favorable net clinical benefit on DCA. Calibration curves showed close concordance between predicted risk and the observed outcomes. SHAP analysis highlighted Septin9 methylation as the most influential variable in the predicting model. Threshold values of 37.3 and 67.1 points were identified as optimal cutoffs for high-risk diseases and CRC discrimination, respectively.

**Conclusions:**

We developed and validated two ML-based models integrating Septin9 methylation with routine serum biomarkers for early detection and differentiation of CRC. These models show potential as non-invasive clinical decision-support tools to facilitate individualized risk assessment and support clinical management in patients undergoing evaluation for colorectal neoplasia.

## Introduction

With rapid economic development and evolving lifestyle patterns, the global burden of colorectal cancer (CRC) has continued to rise. Globally, CRC remains one of the most prevalent malignancies-ranking third in incidence after lung and breast cancer, and second in cancer-related mortality, surpassed only by lung cancer. According to the World Health Organization’s GLOBOCAN 2022 report (Bray et al., 2024), approximately 1.92 million new CRC cases and 904,000 related deaths occurred worldwide in that year alone. Western regions such as Europe, Australia/New Zealand, and North America exhibit the highest incidence, while East Asian countries like China and Japan are experiencing a notable increase (Sung et al., 2021). In China, CRC has become the second most frequently diagnosed cancer, with an estimated 555,000 new cases in 2022, accounting for 12.2% of all malignancies (Zheng et al., 2022).

CRC develops through a complex, multi-step process that typically progresses from histologically normal mucosa to structurally altered but benign adenomatous polyps, and eventually to invasive adenocarcinoma as cellular atypia intensifies and tumor cells breach the basement membrane (Giacometti, Gusella & Cassaro, 2023; Vanoli et al., 2023). Its development is influenced by a range of environmental, metabolic, and genetic factors, including aging, high-fat and low-fiber diets, obesity, smoking, excessive alcohol consumption, chronic intestinal inflammation, and hereditary syndromes such as Familial Adenomatous Polyposis (FAP) and Hereditary Non-Polyposis Colorectal Cancer (HNPCC) (Ip et al., 2025; Goudarzi et al., 2024). Importantly, early-stage CRC often manifests with subtle or non-specific symptoms (Hilal et al., 2025), making it prone to misdiagnosis or delayed detection, which significantly impacts prognosis and quality of life. Consequently, effective strategies for early identification of individuals at increased risk of clinically significant colorectal neoplasia are essential for improving patient outcomes and optimizing healthcare resource utilization.

Current diagnostic approaches for colorectal neoplasms generally fall into invasive or noninvasive categories. Colonoscopy continues to serve as the gold standard (Nooredinvand & Poullis, 2022), offering direct visualization of the colorectal mucosa with the ability to detect, remove and biopsy lesion in real-time. However, its clinical use is limited by procedural discomfort, the need for bowel preparation, risks such as bleeding or perforation, and substantial time and financial costs (Patel & Dominitz, 2024). By contrast, noninvasive stool- and blood-based tests are better tolerated by  patients. The fecal occult blood test (FOBT), for example, is cost-effective, easy to administer, and suitable for population-level screening (Nur et al., 2025), but its diagnostic value is compromised. Bleeding from benign conditions like hemorrhoids or inflammatory bowel disease may result in false positives, while non-bleeding CRCs and precancerous polyps can lead to false negatives (Gómez-Molina et al., 2024; Miles, Rodrigues & Sevdalis, 2013). Classical tumor markers including carcinoembryonic antigen (CEA), CA199, and CA724 are routinely used but have limited specificity because elevated levels may also occur in other gastrointestinal cancers or benign conditions (Wu et al., 2025). Although circulating tumor cells (CTCs) and circulating tumor DNA (ctDNA) represent promising emerging biomarkers, current barriers such as high cost, suboptimal sensitivity in early disease, and technical complexity hinder their widespread application (Dawood et al., 2023; Hu et al., 2022).

Among noninvasive biomarkers, DNA methylation has gained substantial attention. As a major epigenetic mechanism, it involves the transfer of a methyl group to CpG dinucleotides, frequently located within gene promoters (Zheng et al., 2025). While CpG islands remain largely unmethylated in normal tissues, aberrant methylation in cancer can silence tumor-suppressor genes, alter cellular homeostasis, and ultimately promote malignant transformation  (Zheng et al., 2025; Ji et al., 2025). Several methylation-based biomarkers have been validated for the early detection of gastrointestinal tumors (Draškovič et al., 2024; Liu et al., 2024). Genes such as Septin9, SDC2, IRF4, IKZF1, and BCAT1 exhibit cancer-associated methylation patterns relevant to CRC onset and progression (Liu et al., 2024; Mitchell et al., 2016; Mitchell et al., 2014). Notably, Septin9 methylation remains the first-and currently the only-FDA-approved single-gene blood-based methylation marker for CRC screening (Ma et al., 2019). Its methylation level increases significantly with disease progression, making it a compelling biomarker for early detection. Nevertheless, discrepancies in diagnostic accuracy persist across studies, particularly when assessing early-stage CRC or precancerous adenomas. For instance, a prospective clinical study by Church et al. reported sensitivity of only 48.2% for CRC and 11.2% for adenomas (Church et al., 2014), underscoring a high risk of missed diagnoses. Additionally, relatively few studies have focused on the role of Septin9 methylation in differentiating CRC from other colorectal lesions across disease stages.

Machine learning (ML) offers a promising approach to address these challenges by integrating multi-dimensional clinical and laboratory data to capture complex relationships beyond traditional single-marker analyses.  Rather than focusing solely on binary disease detection, ML-based models can facilitate individualized risk stratification and support clinical decision-making, particularly in determining which patients may benefit most from timely evaluation. Therefore, the aim of this study was to develop and validate interpretable ML-based models to support early detection and differentiation of CRC, and to assist risk stratification and colonoscopy decision-making in clinical practice.

## Materials & Methods

### Study participants

**Figure 1:**
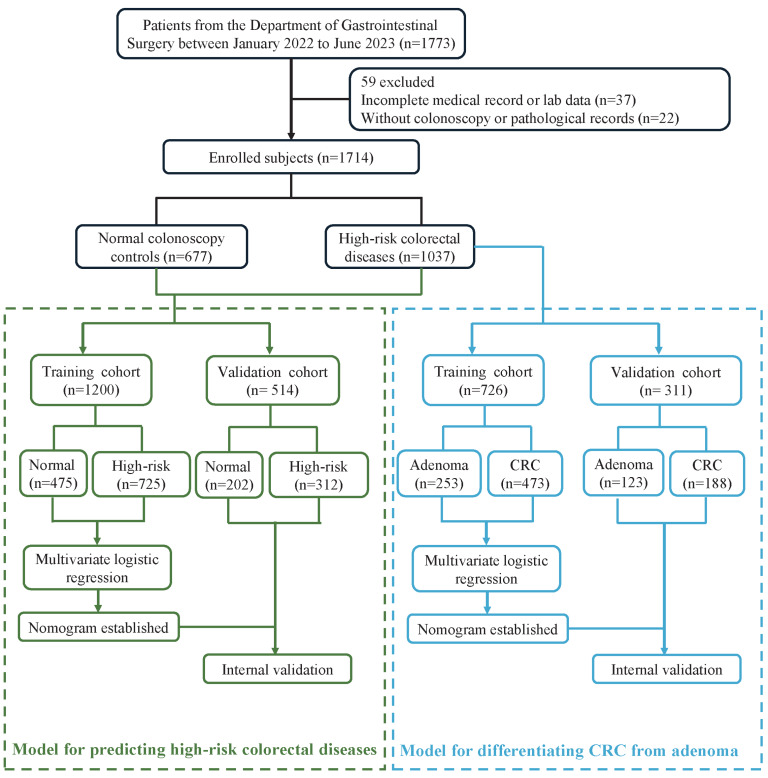
Workflow of this study. CRC, colorectal cancer. High-risk colorectal diseases include patients with colorectal adenoma or CRC confirmed by colonoscopy and histopathology. Normal colonoscopy controls refer to participants with normal colonoscopy findings and no evidence of colorectal neoplasia.

This retrospective study included 1,714 outpatients or inpatients from the Department of Gastrointestinal Surgery at Ruijin Hospital, Shanghai Jiaotong University School of Medicine, between January 2022 and June 2023. All participants provided written informed consent and received colonoscopy examinations, with histopathological assessments performed when clinically indicated. Pathology reports—including tumor location, histological classification, grade of differentiation, and the presence of metastasis—were independently reviewed by two senior pathologists. Advanced adenoma was defined as adenoma exhibiting at least one of the following characteristics: high-grade dysplasia or a size of ≥10 mm. Normal colonoscopy controls were identified based on colonoscopy findings showing no hyperplastic or adenomatous polyps. Demographic and clinical data were retrieved from electronic medical records. The study was reviewed and approved by the Ethical Committee of the Ruijin Hospital, Shanghai Jiaotong University School of Medicine (Ethics approval number: 2020-384). A schematic representation of participant enrollment and the study workflow is shown in Fig. 1.

### Indicators detection

Complete blood count and C-reactive protein (CRP) were performed on Mindray BC-7500 automatic hematology analyzer. Serum tumor indicators including CEA, CA199 and CA724 were analyzed by Roche Cobas E801 chemiluminescent immunoassay system. For values that exceeded analytic measurement ranges, the upper or lower detectable limits were recorded accordingly. Moreover, due to the presence of several extreme high values in the CEA data, a logarithmic transformation (log_10_CEA) was applied to improve its distribution characteristics. FOBT was conducted using a guaiac-based test strip according to standard procedures. Due to its non-routine application in the clinical management of outpatients with normal colonoscopy findings or adenomas, FOBT data were available only for the CRC subgroup and were therefore not considered as candidate variables for model development, but for descriptive purposes only.

The methylation status of Septin9 was determined using a commercial Septin9 Methylation Assay Kit (BioChain Science and Technology, Inc.), following the manufacturer’s protocol. Briefly, 10 mL of peripheral blood was collected using cell-free DNA preservation tubes (CWBIO, Jiangsu, China). Plasma was separated through double centrifugation at 1,500× g for 12 min and subjected to cfDNA extraction. DNA concentration was assessed using a Qubit 3.0 fluorometer (Thermo Fisher Scientific). A total of 5–20 ng of plasma-derived DNA was subjected to bisulfite conversion, followed by real-time polymerase chain reaction (PCR) on a 7500 Fast Real time PCR System (Applied Biosystem) to detect methylated Septin9, with ACTB serving as the internal reference gene. The thermal cycling conditions were as follows: activation at 94 °C for 20 min; 45 cycles at 62 °C for 5 s, 55.5 °C for 35 s, and 93 °C for 30 s; and cooling at 40 °C for 5 s. The methylation status of the Septin9 was determined based on the fluorescence signals and cycle threshold (Ct) values. A Ct value ≤ 41 was considered Septin9 methylation positivity. For samples where no amplification signal was detected, a default Ct value of 42 cycles was assigned. Each independent run included both positive and negative external controls to ensure assay reliability. For descriptive analyses, Septin9 methylation status was presented as a binary variable (positive/negative) according to the manufacturer’s recommended Ct threshold. For model construction, the quantitative Ct value of Septin9 was used as a continuous variable to preserve information content and improve discriminative performance.

### Machine learning model construction and evaluation

In the initial model exploration phase, we evaluated multiple machine learning algorithms, including logistic regression, random forest, support vector machine (SVM), and extreme gradient boosting (XGBoost), to compare the performance (Fig. S1). All models were constructed and internally validated using the same training and validation datasets. After comparing model performance in the validation cohort based on discrimination (AUC), calibration, and clinical interpretability, we selected logistic regression as the final algorithm for reporting to provide a more interpretable nomogram for clinical use. Two ML–based predictive models were then constructed: one for distinguish high-risk colorectal diseases from normal colonoscopy controls, and the other for differentiating CRC from adenoma (Fig. 1). All participants were randomly divided into training and validation cohorts with a 7:3 ratio. The training dataset was used for model development, while the validation dataset was used for internal validation. Univariate logistic regression analyses were conducted to identify variables significantly associated with either high-risk colorectal diseases or CRC. Predictors with *p* < 0.05 were subsequently included in multivariable logistic regression models using standard regression modeling strategies. Odds ratio (OR) with 95% confidence intervals (CIs) were calculated for each selected variable. Based on the final selected predictors, we constructed nomograms and SHapley Additive exPlanations (SHAP) analysis to quantify and visualize the contribution of individual features. Model performance was evaluated through receiver operating characteristic (ROC) curves, calibration plots, and decision curve analysis (DCA) to assess discriminatory power, calibration accuracy, and clinical utility, respectively. Comprehensive diagnostic accuracy metrics, including sensitivity, specificity, predictive values, likelihood ratios, Matthews correlation coefficient (MCC), and confusion matrices were also analyzed where necessary.

### Statistical analysis

Continuous variables were reported as medians with interquartile ranges (IQRs), and categorical data as counts and percentages. All data were analyzed using SPSS version 26.0 (SPSS Inc., Chicago, IL, USA). Group comparisons for continuous variables were performed using Mann–Whitney U or Kruskal–Wallis tests, whereas categorical variables were compared using Pearson’s chi-square test. Spearman correlation coefficients were calculated for correlation analyses, with two-tailed *p* values < 0.05 considered statistically significant. Data visualization was performed using GraphPad Prism 8.0 and R software version 4.4.0 (R Core Team, 2024). All diagnostic accuracy metrics and confusion matrix were calculated using R software version 4.4.0.

## Results

### Baseline characteristics of the participants

A total of 1,714 outpatients or inpatients from the Department of Gastrointestinal Surgery who underwent colonoscopy were included in this study. Among them, 1,037 were diagnosed with high-risk colorectal diseases—including adenomas and CRC—based on pathological confirmation, while the remaining 677 individuals served as normal colonoscopy controls. All relevant indicators of these participants were collected from medical records. The baseline characteristics of the participants are summarized in Table 1. Significant difference was observed in age, gender, Hb, CRP, CEA, CA199 and Septin9 methylation between the two groups (*p* < 0.05). The high-risk colorectal diseases group exhibited a markedly higher median age (62 *vs.* 50 years) with a much higher proportion of individuals over 60 (60.2% *vs.* 21.1%). Additionally, a significant male predominance was noted in the high-risk colorectal diseases group (63.8% *vs.* 55.5%). Meanwhile, high-risk colorectal diseases patients exhibited significantly elevated levels of CRP, CEA, CA199, along with a higher positive rate of Septin9 methylation, while their Hb levels were notably lower (*p* <0.001). Neither CA724 levels nor their abnormal rates differed significantly between the two groups (*p* = 0.712).

**Table 1:** Baseline characteristics of participants in this study.

**Characteristics**	**Overall**	**Normal colonoscopy controls**	**High-risk colorectal** **diseases**	***p*** **value**
	**(*n* = 1,714)**	**(*n* = 677)**	**(*n* = 1,037)**	
**Age, years**	58.0 (48.0, 67.0)	50.0 (42.0, 58.0)	62.0 (54.0, 70.0)	**<0**.**001**
≥60 year	767 (44.7%)	143 (21.1%)	624 (60.2%)	
<60 year	947 (55.3%)	534 (78.9%)	413 (39.8%)	
**Gender**				**0**.**001**
Male	1,038 (60.6%)	376 (55.5%)	662 (63.8%)	
Female	676 (39.4%)	301 (44.5%)	375 (36.2%)	
**Hemoglobin, g/L**	137 (125, 150)	143 (133, 155)	132 (118, 145)	**<0**.**001**
**CRP, mg/L**	1.20 (0.80, 3.10)	0.90 (0.50, 1.60)	2.00 (1.00, 5.00)	**<0**.**001**
**CEA, ng/ml**	1.79 (1.07, 3.23)	1.27 (0.85, 1.97)	2.41 (1.38, 5.17)	**<0**.**001**
>5 ng/ml	276 (16.1%)	8 (1.18%)	268 (25.8%)	
≤5 ng/ml	1,438 (83.9%)	669 (98.8%)	769 (74.2%)	
**CA199, U/ml**	7.90 (4.60, 14.4)	7.10 (4.50, 11.1)	8.90 (4.70, 18.8)	**<0**.**001**
>25 U/ml	207 (12.1%)	19 (2.81%)	188 (18.1%)	
≤25 U/ml	1,507 (87.9%)	658 (97.2%)	849 (81.9%)	
**CA724, U/ml**	2.12 (1.49, 4.57)	2.15 (1.47, 4.46)	2.08 (1.50, 4.69)	0.712
>8.2 U/ml	232 (13.5%)	78 (11.5%)	154 (14.9%)	
≤8.2 U/ml	1,482 (86.5%)	599 (88.5%)	883 (85.1%)	
**Septin9**				**<0**.**001**
Positive	396 (23.1%)	10 (1.48%)	386 (37.2%)	
Negative	1,318 (76.9%)	667 (98.5%)	651 (62.8%)	

**Notes.**

Continuous variables are presented as medians (interquartile ranges, IQR), and categorical variables are reported as frequency (percentage). High-risk colorectal diseases include patients with colorectal adenoma or CRC confirmed by colonoscopy and histopathology. Normal colonoscopy controls refer to participants with normal colonoscopy findings and no evidence of colorectal neoplasia. Statistically significant values (*p* < 0.05) are shown in bold.

### Construction and validation of a model for predicting high-risk colorectal diseases

All participants were randomly divided into training and validation cohorts at a 7:3 ratio. The seven biomarkers mentioned above were used for univariate logistic regression on the training cohort, and variables with a *p* value < 0.1 were included in the multivariate logistic regression analysis. As shown in Table 2, gender, age, Septin9 methylation, Hb, CRP, and CEA are identified as independent predictors of high-risk colorectal diseases. In univariable analysis, gender was not a significant predictor (*p* = 0.088), while CA199 showed a significant association with the outcome (*p* < 0.001). However, in the multivariate analysis, the results were reversed. This shift indicates that the apparent effect of gender was confounded by other variables, such as age. After statistically isolating its influence, male gender was revealed to be a strong standalone risk factor, associated with a 147% increased risk. Conversely, CA199 lost its significance, implying that it does not provide unique predictive information beyond what is already captured by stronger covariates like CEA and Septin9 methylation.

**Table 2:** Univariable and multivariate logistic regression analysis for high-risk colorectal diseases prediction model in the training cohort.

**Indicators**	**Normal colonoscopy controls**	**High-risk colorectal diseases**	**Univariable logistic regression**		**Multivariate logistic regression**	
	**(** ***n*** **= 475)**	**(** ***n*** **= 725)**	**Adjusted OR (95% CI)**	***p*** ** value**	**Adjusted OR (95% CI)**	***p*** ** value**
**Gender:**			1.228 (0.970–1.554)	0.088	2.474 (1.675–3.656)	**<0**.**001**
Male	270 (56.8%)	448 (61.8%)				
Female	205 (43.2%)	277 (38.2%)				
**Age**	50.0 (43.0, 58.0)	62.0 (54.0, 70.0)	1.083 (1.071–1.096)	**<0**.**001**	1.060 (1.045–1.075)	**<0**.**001**
**Septin9**	42.0 (42.0, 42.0)	42.0 (39.5, 42.0)	0.245 (0.168–0.356)	**<0**.**001**	0.249 (0.166–0.372)	**<0**.**001**
**Hb**	144 (133, 156)	131 (118, 145)	0.960 (0.953–0.967)	**<0**.**001**	0.955 (0.943–0.966)	**<0**.**001**
**CRP**	0.90 (0.50, 1.55)	2.00 (1.00, 5.00)	1.193 (1.135–1.255)	**<0**.**001**	1.068 (1.013–1.126)	**0**.**014**
**log**_**10**_ **CEA**	0.12 (−0.05, 0.30)	0.37 (0.13, 0.69)	11.211 (7.410–16.962)	**<0**.**001**	4.213 (2.562–6.927)	**<0**.**001**
**CA199**	7.10 (4.60, 11.2)	8.60 (4.60, 18.0)	1.030 (1.018–1.041)	**<0**.**001**		

**Notes.**

Continuous variables are presented as medians (interquartile ranges, IQR), and categorical variables are reported as frequency (percentage). Odds ratios (ORs) are presented with 95% confidence intervals (CIs). High-risk colorectal diseases include patients with colorectal adenoma or CRC confirmed by colonoscopy and histopathology. Normal colonoscopy controls refer to participants with normal colonoscopy findings and no evidence of colorectal neoplasia. Statistically significant values (*p* < 0.05) are shown in bold.

**Figure 2:**
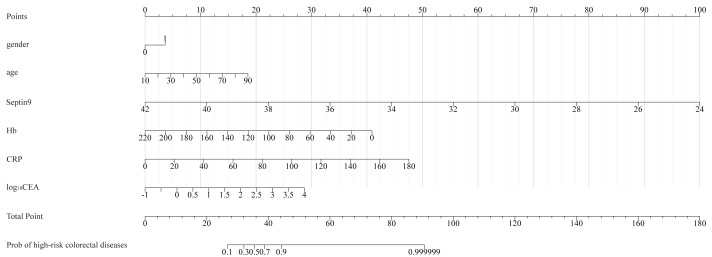
Nomogram for predicting high-risk colorectal diseases. Hb, hemoglobin; CRP, C-reactive protein; CEA, carcinoembryonic antigen.

Figure 2 showed the nomogram for distinguishing patients with high-risk colorectal diseases from normal colonoscopy controls. Male gender contributes 3.6 points to the risk score. For every 10-year increase in age, the risk score increases by 2.3 points. A decrease of 1 unit in the Septin9 methylation Ct value adds 5.6 points, while a decrease of 10 g/L in Hb adds 1.9 points. Furthermore, an increase of 10 units in CRP raises the score by 2.6 points, and a 1-unit increase in log_10_CEA contributes an additional 5.7 points to the risk assessment. Based on our predictive model, an individualized total risk score can be calculated for each patient. For example, a 65-year-old male patient presented with the following test results:, Hb = 120 g/L, CRP = 5 mg/L, and CEA = 2.0 ng/mL, Septin9 methylation Ct value = 35. According to the nomogram, age (65 years) contributes approximately 12.8 points, male sex contributes 3.6 points, a Septin9 Ct value of 35 contributes 38.9 points, Hb of 120 g/L contributes 18.6 points, CRP of five mg/L contributes 1.3 points, and log_10_CEA of 0.3 contributes 7.5 points. The sum of these components yields a total risk score of approximately 82.7 points. An optimal risk threshold of 37.3 points was established by correlating the nomogram scores with clinical outcomes, corresponding to the maximum Youden Index in the ROC analysis (Fig. S2). The predictive accuracy of the nomogram at this threshold is detailed in Table 3, which confirms its robust diagnostic performance and stability. Consequently, a total risk score exceeding 37.3 points classifies an individual as high-risk, warranting referral for further diagnostic evaluation.

**Table 3:** Comprehensive performance metrics of the nomograms for both models.

**Category**	**Metric**	**Prediction model**		**Differentiation model**	
		**Training (*n* = 1,200)**	**Validation (*n* = 514)**	**Training (*n* = 726)**	**Validation (*n* = 311)**
**Confusion Matrix**	**TP**	533	227	361	141
	**FP**	49	23	48	24
	**FN**	192	85	112	47
	**TN**	426	179	205	99
**Performance**	**AUC**	0.879	0.881	0.845	0.836
	**Sensitivity**	0.735	0.728	0.763	0.750
	**Specificity**	0.897	0.886	0.810	0.805
	**PPV**	0.916	0.908	0.883	0.855
	**NPV**	0.689	0.678	0.647	0.678
	**LR+**	7.127	6.390	4.023	3.844
	**LR-**	0.295	0.307	0.292	0.311
	**MCC**	0.618	0.600	0.551	0.544

**Notes.**

TP True positive FP False positive FN False negative TN True negative AUC Area under the receiver operating characteristic curve PPV Positive predictive value NPV Negative predictive value LR+ Positive likelihood ratio LR- Negative likelihood ratio MCC Matthews Correlation Coefficient

**Figure 3:**
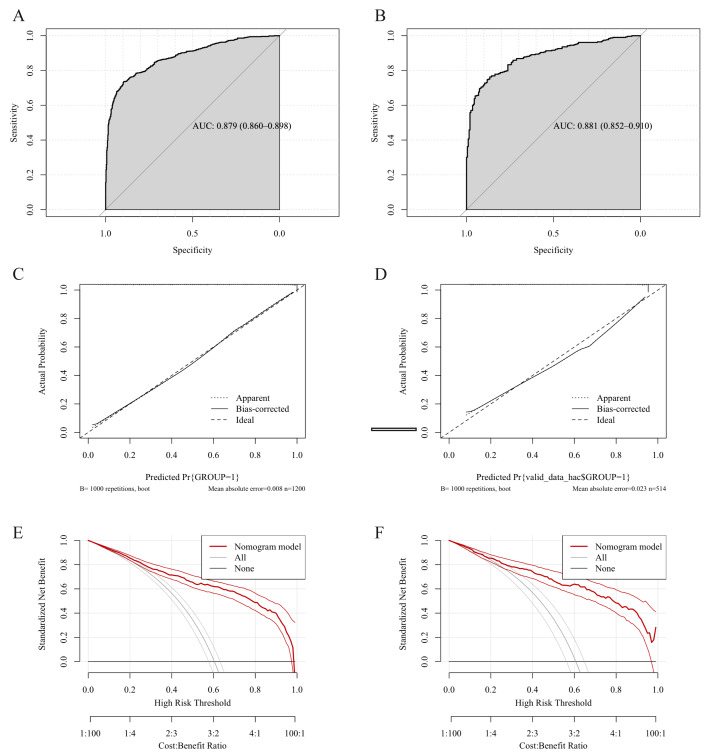
Validation of the high-risk colorectal diseases prediction model. Receiver operating characteristic (ROC) curves, calibration plots and decision curve analysis (DCA) in the training (A, C, E) and validation (B, D, F) datasets.

ROC curves, calibration curves and DCA were utilized to evaluate the prediction model. As shown in Figs. 3A and 3B, the model demonstrates robust and generalizable performance with high, consistent AUC values of training (0.879, 95% CI [0.860–0.898]) and validation (0.881, 95% CI [0.852–0.910]) datasets. Figures 3C and 3D demonstrate excellent model calibration in both training and validation cohorts, with nearly identical and very low mean absolute errors (0.008 and 0.023, respectively), indicating highly reliable predicted probabilities that closely match actual outcomes. The DCA curves (Figs. 3E and 3F) confirm the model’s strong clinical utility, offering a greater net benefit than the treat-all strategy across a wide threshold probability range (0.01 to 0.60) in both training and validation cohorts.

### Septin9 methylation as a key predictor and its association with CRC progression

**Figure 4:**
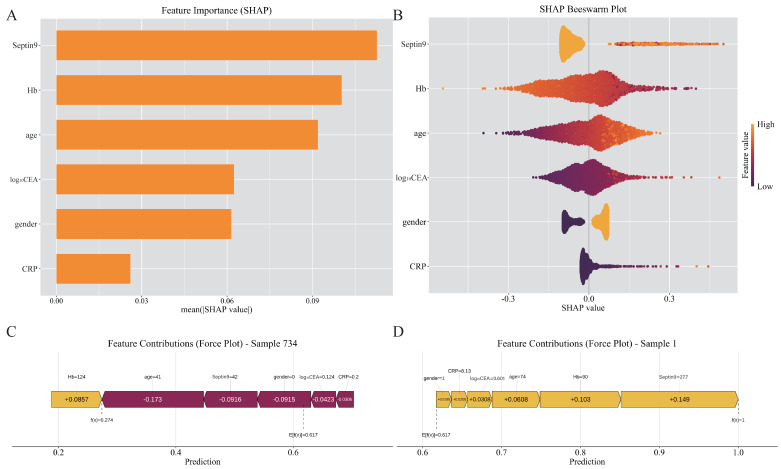
SHAP interpretation of feature contributions in the high-risk colorectal diseases prediction model. (A) Bar plot of mean SHAP values, ranking features by their average importance across the dataset. (B) SHAP summary plot showing the impact of each feature on the model’s output. Each dot represents a single prediction. The *X*-axis indicates the SHAP value (impact on model output), while the *Y*-axis ranks the features in descending order based on their average impact on model predictions. The color represents the original feature value (purple = low, yellow = high). (C) SHAP force plot for a healthy individual correctly predicted as negative. (D) SHAP force plot for an individual CRC patient correctly predicted as positive.

We then investigated the contribution of each indicator in the model. Figures 4A and 4B present the summary SHAP values, highlighting the relative contribution of each feature to the model output. Features are ranked in descending order of their mean absolute impact on prediction magnitude. Notably, Septin9 methylation emerged as the most influential variable in shaping individual predictions. To interpret the model’s prediction for an individual patient, we employed SHAP force plots (Figs. 4C & 4D). Younger age, low levels of tumor biomarkers and negative Septin9 methylation push the model prediction toward the negative class. For this specific 41-year-old female patient, the model output a raw prediction value of *f*(*x*) = 0.274, significantly lower than the model’s base value (0.621). These visualizations clearly demonstrate that Septin9 methylation plays a vital role in shaping diagnostic outcomes on a per-patient basis.

**Table 4:** The impact of Septin9 methylation on other indicators among the three groups of participants.

**Characteristic**	**Normal colonoscopy controls (*n* = 677)**		***p*** **value**	**Adenomas (*n* = 376)**		***p*** **value**	**CRC (*n* = 661)**		***p*** **value**
	**Septin9+ (*n* = 10)**	**Septin9- (*n* = 667)**		**Septin9+ (*n* = 116)**	**Septin9- (*n* = 260)**		**Septin9+ (*n* = 270)**	**Septin9- (*n* = 391)**	
**Age, years**	60.0 (50.0, 70.0)	50.0 (42.0, 57.0)	**0.021**	58.0 (52.0, 68.0)	59.0 (52.0, 64.0)	0.087	66.0 (57.0, 73.0)	65.0 (57.0, 72.0)	0.248
≥60 year	5 (50.0%)	138 (20.7%)		54 (46.6%)	120 (46.2%)		187 (69.3%)	263 (67.3%)	
<60 year	5 (50.0%)	529 (79.3%)		62 (53.4%)	140 (53.8%)		83 (30.7%)	128 (32.7%)	
**Gender**			0.525			0.565			0.124
Male	7 (70.0%)	369 (55.3%)		84 (72.4%)	179 (68.8%)		173 (64.1%)	226 (57.8%)	
Female	3 (30.0%)	298 (44.7%)		32 (27.6%)	81 (31.2%)		97 (35.9%)	165 (42.2%)	
**Hb, g/L**	150 (142, 164)	143 (133, 155)	0.110	137 (128, 151)	144 (131, 153)	**0**.**018**	122 (105, 137)	128 (115, 140)	**<0**.**001**
**CRP, mg/L**	1.02 (0.36,1.54)	1.02 (0.36,1.54)	0.891	1.23 (0.74, 2.41)	1.10 (1.00, 2.58)	0.316	4.00 (1.44, 9.47)	2.00 (1.00, 5.00)	**<0**.**001**
**CEA, ng/ml**	1.29 (0.94, 1.55)	1.27 (0.85, 1.97)	0.953	1.84 (1.19, 2.88)	1.48 (0.94, 2.27)	**0**.**002**	4.84 (2.46, 25.7)	2.73 (1.63, 5.49)	**<0**.**001**
>5 ng/ml	0 (0.00%)	8 (1.20%)		8 (6.90%)	13 (5.00%)		134 (49.6%)	113 (28.9%)	
≤5 ng/ml	10 (100%)	659 (98.8%)		108 (93.1%)	247 (95.0%)		136 (50.4%)	278 (71.1%)	
**CA199, U/ml**	6.05 (4.05,8.70)	7.10 (4.50, 11.1)	0.278	7.95 (5.25, 13.7)	5.95 (3.88,9.93)	**0**.**001**	14.3 (6.70, 46.7)	9.50 (4.80,20.2)	**<0**.**001**
>25 U/ml	0 (0.00%)	19 (2.85%)		10 (8.62%)	11 (4.23%)		94 (34.8%)	73 (18.7%)	
≤25 U/ml	10 (100%)	648 (97.2%)		106 (91.4%)	249 (95.8%)		176 (65.2%)	318 (81.3%)	
**FOBT**									0.571
Positive							152 (56.3%)	230 (58.8%)	
Negative							118 (43.7%)	161 (41.2%)	
**Cancer type**									**0**.**001**
Infiltrative							10 (3.70%)	4 (1.02%)	
Ulcerative							180 (66.7%)	224 (57.3%)	
Protrude							80 (29.6%)	163 (41.7%)	
**TNM Stage**									**<0**.**001**
I							9 (3.33%)	22 (5.63%)	
II							108 (40.0%)	212 (54.2%)	
III							92 (34.1%)	130 (33.2%)	
IV							61 (22.6%)	27 (6.91%)	
**Histological grade**									**0**.**005**
Low							63 (23.3%)	63 (16.1%)	
Moderate							203 (75.2%)	308 (78.8%)	
High							4 (1.48%)	20 (5.12%)	
**Location**									0.342
Left colon							92 (34.1%)	136 (34.8%)	
Rectum							111 (41.1%)	167 (42.7%)	
Right colon							60 (22.2%)	70 (17.9%)	
Transverse colon							7 (2.59%)	18 (4.60%)	
**Metastasis**									**<0**.**001**
Node							85 (31.5%)	132 (33.8%)	
Organ							23 (8.52%)	9 (2.30%)	
Both							44 (16.3%)	17 (4.35%)	
None							118 (43.7%)	233 (59.6%)	
**Family history**									**0**.**027**
Positive							46 (17.0%)	96 (24.6%)	
Negative							224 (83.0%)	295 (75.4%)	

**Notes.**

Continuous variables are presented as medians (interquartile ranges, IQR), and categorical variables are reported as frequency (percentage). Normal colonoscopy controls refer to participants with normal colonoscopy findings and no evidence of colorectal neoplasia. Statistically significant values (*p* < 0.05) are shown in bold.

Since Septin9 methylation was demonstrated as the most substantial contributing feature in the model, we then conducted a stratified analysis based on methylation status (Table 4). Among the 1,037 high-risk colorectal diseases patients, 376 were diagnosed by colonoscopy with adenomas and 661 with CRC. The positivity rate of Septin9 methylation was lowest in normal colonoscopy controls (1.48%) and progressively increased in adenoma and CRC patients (30.85% and 40.85%, respectively). Among the normal colonoscopy controls, individuals with positive Septin9 methylation are generally older (*p* = 0.021), and no significant correlations have been found between this biomarker and other indicators. In patients with adenoma, those who are Septin9 methylation positive tend to exhibit lower hemoglobin levels, along with elevated CEA and CA199 levels (*p* < 0.05). As for CRC patients, significant differences are observed between the other serum biomarkers among Septin9 methylation positive and negative subsets (*p* < 0.001). Septin9 methylation positive CRC patients are more likely to develop into infiltrative and ulcerative types of CRC, with a significantly higher positivity rate observed in stages III and IV. These patients also show a greater prevalence of low grade histology, a reduced proportion of high-grade tumors, and significant higher rates of organ metastasis, as well as concurrent lymph node and organ involvement. Interestingly, a higher proportion of Septin9 methylation negative CRC patients reported a family history of cancer.

Moreover, we evaluated the stage-specific performance of Septin9 alone by comparing CRC patients at each TNM stage against normal-colonoscopy controls (Table S1). A clear trend of improvement was observed with disease progression. The AUC increased progressively from 0.638 in Stage I to 0.844 in Stage IV, and sensitivity improved substantially from 29.0% to 69.3%. Notably, high specificity (>98.5%) was preserved across all stages. This marked sensitivity gap in early-stage diseases is precisely the limitation our integrated nomogram aims to overcome.

### Construction and validation of a model for differentiating CRC from adenoma

Similarly, all high-risk colorectal diseases patients (*n* = 1,037) were randomly divided into training and validation cohorts at a 7:3 ratio. Multivariate logistic regression identified age, Septin9 methylation, Hb, CRP and CEA are identified as independent predictors of CRC (Table 5). Septin9 methylation and CA199, though significant in univariable analysis, lost independence in the multivariate model, indicating their discriminative ability between CRC and adenoma is superseded by stronger predictors like CEA. Moreover, the association of gender was confounded by other variables, confirming it lacks independent predictive value for this differentiation.

**Table 5:** Univariable and multivariate logistic regression analysis for differentiating CRC from adenomas in the training cohort.

**Parameters**	**Adenomas**	**CRC**	**Univariable logistic regression**		**Multivariate logistic regression**	
	**(** ***n*** **= 253)**	**(** ***n*** **= 473)**	**Adjusted OR (95% CI)**	***p*** ** value**	**Adjusted OR (95% CI)**	***p*** ** value**
**Gender:**			0.706 (0.507–0.977)	**0**.**036**		
Male	177 (70.0%)	294 (62.2%)				
Female	76 (30.0%)	179 (37.8%)				
**Age**	58.0 (52.0, 65.0)	66.0 (57.0, 72.0)	1.049 (1.034–1.064)	**<0**.**001**	1.024 (1.006–1.042)	**0**.**007**
**Septin9**	42.0 (40.3, 42.0)	42.0 (38.3, 42.0)	0.785 (0.723–0.851)	**<0**.**001**	0.929 (0.824–1.028)	0.141
**Hb**	142 (131, 153)	127 (111, 138)	0.957 (0.948–0.967)	**<0**.**001**	0.964 (0.954–0.975)	**<0**.**001**
**CRP**	1.10 (1.00, 2.60)	3.00 (1.00, 8.00)	1.098 (1.055–1.143)	**<0**.**001**	1.048 (1.011–1.085)	**0**.**011**
**log**_**10**_ **CEA**	0.21 (0.00, 0.38)	0.54 (0.27, 1.02)	12.109 (7.261–20.194)	**<0**.**001**	9.788 (5.529–17.326)	**<0**.**001**
**CA199**	6.40 (4.30, 11.3)	11.3 (5.20, 24.7)	1.017 (1.009–1.026)	**<0**.**001**		
**CA724**	1.99 (1.44, 3.89)	2.20 (1.50, 5.30)	1.014 (0.999–1.030)	0.072		

**Notes.**

Continuous variables are presented as medians (interquartile ranges, IQR), and categorical variables are reported as frequency (percentage). Odds ratios (ORs) are presented with 95% confidence intervals (CIs). Statistically significant values (*p* < 0.05) are shown in bold.

**Figure 5:**
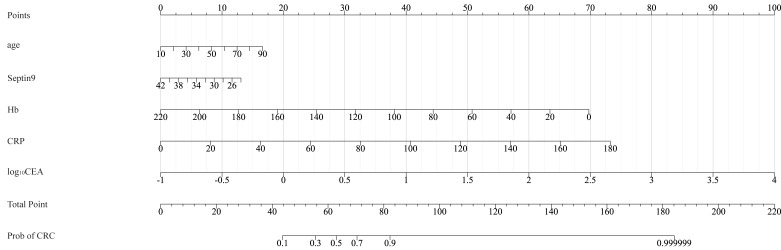
Nomogram for differentiating CRC from adenoma. Hb, hemoglobin; CRP, C-reactive protein; CEA, carcinoembryonic antigen.

The resulting nomogram visualizes the relative contributions of each predictors for differentiating CRC patients from adenoma patients (Fig. 5). For every 10-year increase in age, the risk score increases by 2.1 points. A decrease of 1 unit in the Septin9 methylation Ct value adds 0.7 points, while a decrease of 10 g/L in Hb adds 3.2 points. Furthermore, an increase of 10 units in CRP raises the score by 4.1 points, and a 1-unit increase in log_10_CEA contributes an additional 20 points to the risk assessment. Similarly, based on the correlation of nomogram scores with clinical outcomes, 67.1 points was identified as the optimal risk threshold to identify CRC cases, as it represented the maximum Youden Index in the ROC analysis (Fig. S2). Analysis of the same example patient mentioned in prior with this nomogram showed that age (65 years) contributes approximately 11.4 points, a Septin9 Ct value of 35 contributes 5.1 points, Hb of 120 g/L contributes 31.7 points, CRP of five mg/L contributes 2.0 points, and log_10_CEA of 0.3 contributes 26.0 points. The sum of these components yields a total risk score of approximately 76.2 points. This score further supports a high suspicion of CRC in this patient and underscores the clinical indication for prompt colonoscopy and subsequent management. As shown in Table 3, the nomogram achieves reliable predictive accuracy at this threshold, confirming its clinical validity and generalizability across the evaluated cohort.

**Figure 6:**
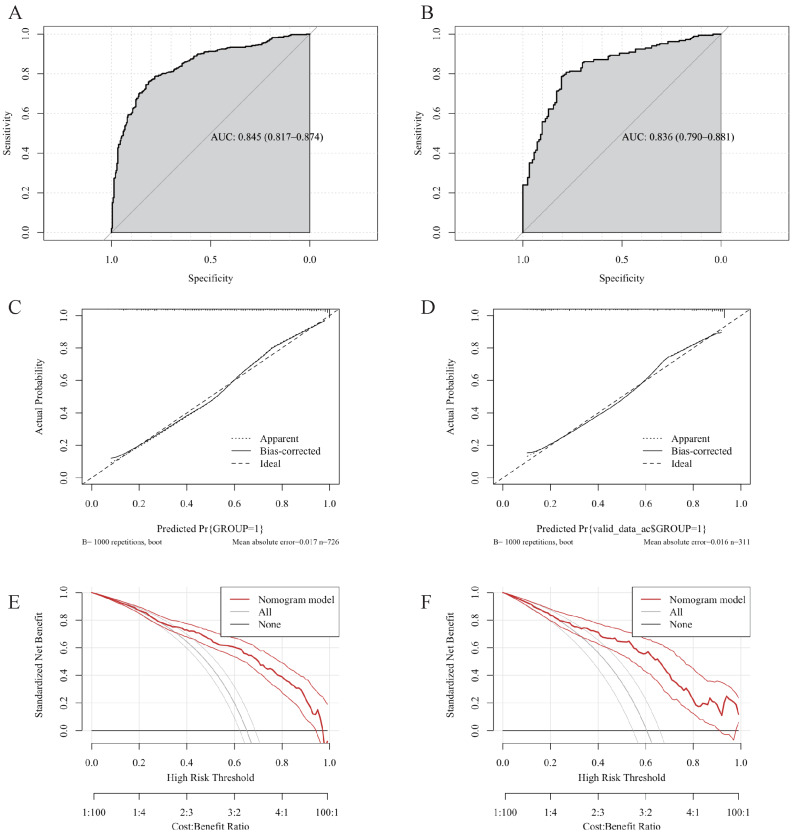
Validation of the CRC differentiation model. Receiver operating characteristic (ROC) curves, calibration plots and decision curve analysis (DCA) in the training (A, C, E) and validation (B, D, F) datasets.

This differentiation model also showed strong discrimination (training AUC 0.845; validation AUC 0.836) and excellent calibration (Mean absolute error 0.017 and 0.016) (Figs. 6A–6D). DCA indicated clear net benefit over the treat-all approach across a broad probability range (0.01–0.65), confirming the model’s robustness and clinical relevance (Figs. 6E and 6F) .

## Discussion

Early-stage CRC remains challenging to identify because disease progression is often asymptomatic or associated with nonspecific clinical manifestations, leading to delayed clinical recognition. Moreover, the considerable genetic and epigenetic heterogeneity of CRC complicates early risk assessment, while current stratification strategies may fail to accurately identify individuals at elevated risk or may direct low-risk individuals toward unnecessary invasive procedures. Additional barriers, including limited access to healthcare resources, patient hesitation toward colonoscopy, and the inherent difficulty of detecting advanced adenomas—further hinder timely intervention. Notably, our study revealed a disproportionately higher rate of advanced adenomas among individuals younger than 60 years (Table 4), underscoring the need for heightened clinical vigilance and potentially earlier diagnostic consideration in selected patients. It should be emphasized, however, that our cohort consisted of outpatients or inpatients from a gastrointestinal surgery department, resulting in substantially higher detection rates of CRC and adenoma than would be expected in population-based screening settings. Consequently, external validation in prospective cohorts—particularly those representing screening or lower-risk populations—is required before broader clinical implementation.

The combined use of multiple biomarkers, particularly DNA methylation markers, has shown substantial promise for enhancing diagnostic sensitivity and specificity in gastrointestinal malignancies. For example, a recently developed six-gene methylation panel demonstrated potential for early gastrointestinal cancer detection (Dai et al., 2023). Similarly, Caraballo et al. (2024) found that a multi-analyte panel incorporating Septin9 methylation alongside IGFBP2, DKK3, and PKM2 improved CRC discrimination. Traditional multi-maker diagnostic approaches often rely on predefined rules or expert interpretation. In contrast, ML algorithms, particularly those designed to handle high-dimensional data, offer the potential to uncover complex relationships and identify feature interactions without predefined assumptions, making it particularly suitable for integrating heterogeneous biomarker data.

From a methodological perspective, strict separation into training, testing, and validation datasets is theoretically optimal for unbiased assessment of model generalizability in ML studies. In the present work, we employed a commonly used “train-validation” split method (7:3) applied in clinical prediction model research. The training cohort was used exclusively for model construction and fitting, and no model restructuring, feature reselection, or parameter adjustment was performed based on validation performance. Although multiple ML approaches, including logistic regression, SVM, random forest, and XGBoost were evaluated during preliminary analyses (Fig. S1), logistic regression was selected for final model reporting because of its interpretability, clinical transparency, and stable performance. This approach also facilitates the development of user-friendly nomograms, enhancing clinical applicability. Both models we constructed in this study exhibited strong discriminative performance (AUC > 0.83) and satisfactory calibration, supporting respectable predictive accuracy. Nonetheless, a modest decrease in AUC between the training and validation cohorts (0.03–0.04) suggests mild overfitting, underscoring the need for external validation using independent datasets. Moreover, decision curve analysis showed a sharp reduction in net clinical benefit when the risk threshold exceeded 0.6, implying diminished effectiveness in identifying individuals with very high risk. Future refinements, such as stratified modeling or incorporation of interaction terms, may help improve performance in this subgroup.

The optimal threshold of 37.3 and 67.1 points derived from the nomogram-based risk indices offer practical tools for clinical triage. Individuals scoring above 37.3 are more likely to harbor high-risk colorectal lesions and should be prioritized for colonoscopy, whereas scores exceeding 67.1 strongly suggest the presence of CRC. These thresholds translate statistical outputs into meaningful, actionable decision rules, effectively bridging predictive analytics and clinical practice. However, given the higher baseline risk in our clinical cohort, the absolute risk associated with these cutoffs would be expected to decrease in lower-prevalence populations, such as general screening settings. Accordingly, external validation and recalibration in target populations represent important subsequent steps toward broader clinical application.

Consistent with previous research, Septin9 methylation was the most influential predictor in the predictive model and showed a strong association with CRC progression (Fig. 4 and Table 4). However, Table S2 indicates that relying solely on clinical baseline factors (such as age and gender) or on the Septin9 marker alone yields limited diagnostic performance. The nomogram constructed by integrating Septin9 with other serum biochemical markers achieved a significant improvement in AUC (from 0.795 to 0.881) and MCC (from 0.456 to 0.615). This demonstrates that while clinical baselines are important, the addition of molecular and laboratory indicators is essential for achieving the high diagnostic precision required for clinical decision-making. Early meta-analyses have documented moderate diagnostic accuracy for the Septin9 assays, with reported sensitivities ranging from 48% to 95.6% and specificities between 79% and 99% across diverse cohorts (Song et al., 2017). Building upon previous work that largely assessed Septin9 as a standalone biomarker, our findings demonstrate that combining Septin9 methylation with routine serum biomarkers yields a gain in accuracy for both differentiating adenoma from CRC and detecting early-stage disease (Table S1, Fig. S2). This approach aligns with the findings of Zou et al. (2024), who achieved an AUC of 0.908 using a combined Septin9–SDC2 methylation panel with serum tumor markers, albeit without ML modeling or subtype-specific analysis. To preserve maximal information and enhance discriminative power, we incorporated the quantitative Ct value of Septin9 as a continuous variable in model construction. Additional analysis replacing Ct values with binary Septin9 status (positive/negative) yielded comparable discrimination, despite binary Septin9 yielded a positive and significant odds ratio in the predictive model (Table S3), indicating that model performance is not critically dependent on its quantitative representation. The reduced contribution of binary Septin9 to CRC differentiation is biologically expected, as most CRC cases are Septin9-positive, limiting its discriminatory value when dichotomized.

Despite these encouraging results, several limitations should be acknowledged. Firstly, the sample size of CRC and adenoma patients was relatively small and derived from a single center, which may restrict generalizability and introduce selection bias compared to multicenter or population-based datasets. To mitigate overfitting, we employed multiple safeguards, including random train–validation splitting, use of a parsimonious and interpretable modeling approach, and comprehensive performance evaluation using AUC, calibration curves, and decision curve analysis. The consistent performance across datasets suggests acceptable internal robustness. Nevertheless, internal validation alone cannot fully establish generalizability, and external validation in independent cohorts remains necessary. Secondly, the biomarker spectrum included in our model was limited. Incorporating additional data modalities, such as radiomic features from computed tomography (CT) imaging, liquid biopsy components (*e.g.*, ctDNA), or gut microbiome profiles may enhance predictive performance. Future work should emphasize multicenter validation, integration of multimodal biomarkers, and refinement of analytical sensitivity through methods such as digital PCR. These efforts will be essential for strengthening clinical applicability and ensuring robust model performance across diverse patient populations.

## Conclusions

In summary, we established and validated two ML-based models integrating Septin9 methylation with multiple routinely available serum biomarkers to identify individuals at high risk for colorectal diseases and to distinguish CRC from adenoma, respectively. Both models exhibited excellent discrimination power, reliable calibration, and meaningful clinical applicability. These results highlight the value of combining ML approaches with epigenetic and laboratory markers for improving early detection and differentiation of CRC, and to assist risk stratification and colonoscopy decision-making in clinical practice. Further validation in larger, prospective, and multicenter cohorts is warranted before broader clinical implementation.

## Supplemental Information

10.7717/peerj.21053/supp-1 Supplemental Information 1 Raw data of participants

10.7717/peerj.21053/supp-2 Supplemental Information 2 ROC curves demonstrating the diagnosis performance of six machine learning algorithms SVM: Support Vector Machine; XGBoost: eXtreme Gradient Boosting; LR: Logistic Regression; KNN: k-Nearest Neighbor.

10.7717/peerj.21053/supp-3 Supplemental Information 3 ROC curves for the optimal threshold s of the two nomograms (A) ROC curves f or 37.3 points as the optimal threshold for predicting high-risk colorectal disease s among training and validation set, respectively. (B) ROC corves for 67.1 points as the optimal threshold for differentiating CRC from adenoma among training and validation set, respectively. Sens: sensitivity; Spec: specificity; AUC: Area under the receiver operating characteristic curve.

10.7717/peerj.21053/supp-4 Supplemental Information 4 Diagnostic performance of Septin9 across different TNM Stages of CRC patients

10.7717/peerj.21053/supp-5 Supplemental Information 5 Comprehensive diagnostic performance of the nomogram and baseline markers in the two models

10.7717/peerj.21053/supp-6 Supplemental Information 6 Multivariate logistic regression models and diagnostic performance based on binary Septin9 status

10.7717/peerj.21053/supp-7 Supplemental Information 7 Code for all tables and figures

## Additional Information and Declarations

### Data Availability

The following information was supplied regarding data availability: The raw data and code are available in the Supplemental Files.
