# Supplementary figures and images for: Development and validation of machine learning-based models integrating Septin9 methylation and serum biomarkers for early detection and differentiation of colorectal cancer
Source: PeerJ. 2026 Mar 31;14:e21053. doi: 10.7717/peerj.21053 (PMC13048225; doi:10.7717/peerj.21053)

ROC Curves for Test Set

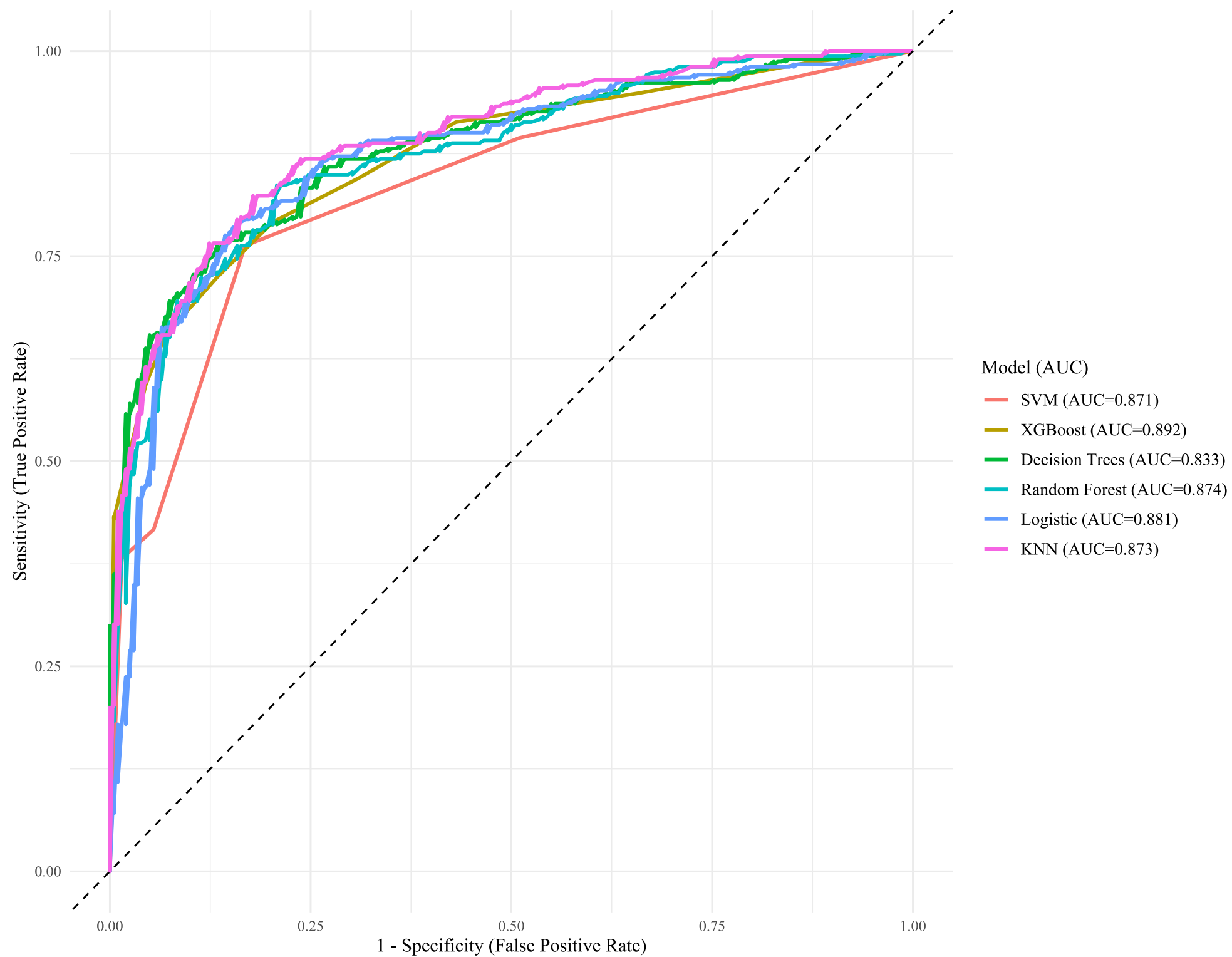

Supplement: Supplemental Information 2 — SVM: Support Vector Machine; XGBoost: eXtreme Gradient Boosting; LR: Logistic Regression; KNN: k-Nearest Neighbor. [file peerj-14-21053-s002.pdf]

A

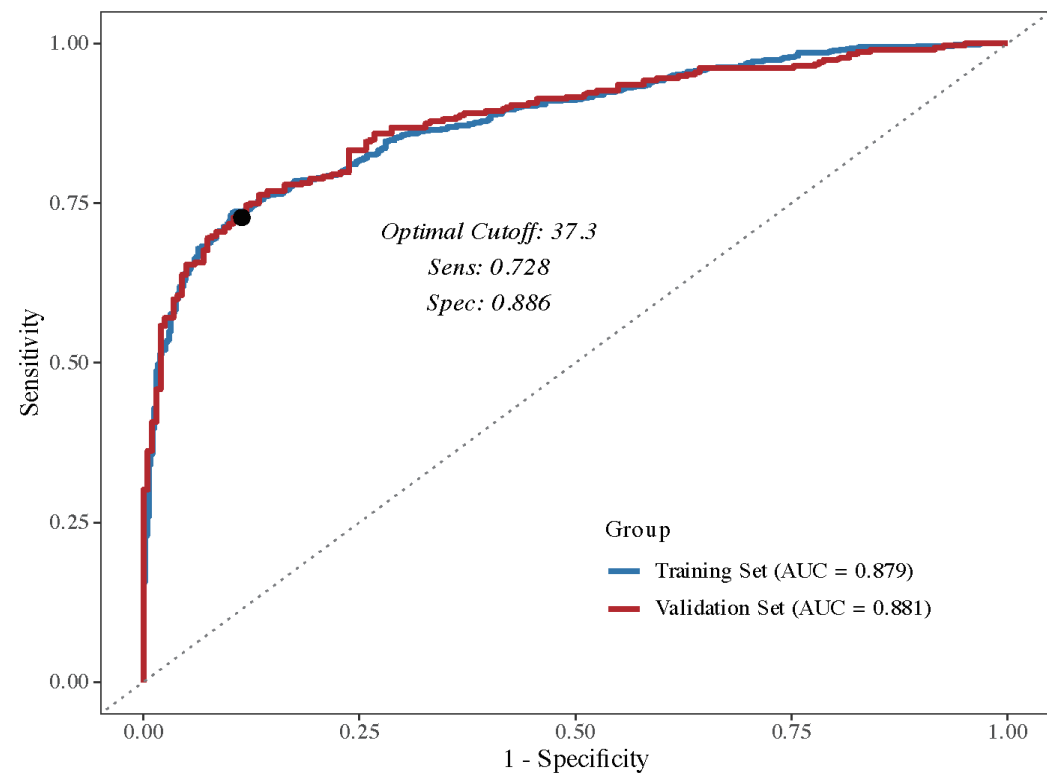

B

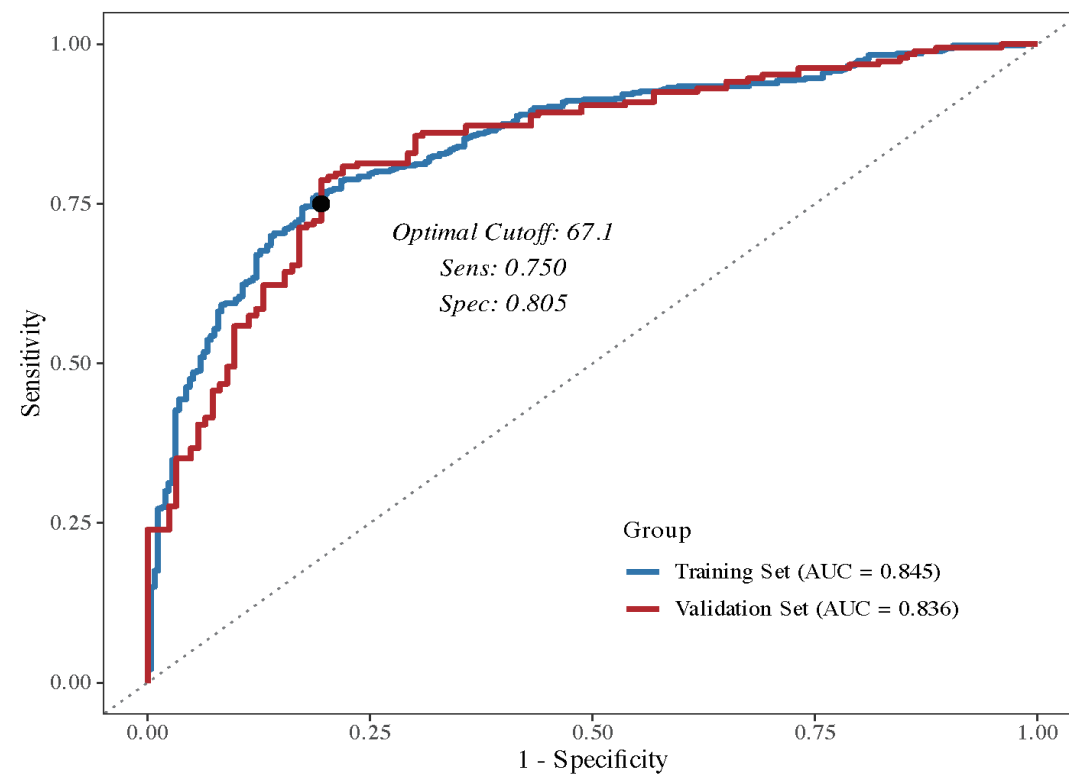

Supplement: Supplemental Information 3 — (A) ROC curves f or 37.3 points as the optimal threshold for predicting high-risk colorectal disease s among training and validation set, respectively. (B) ROC corves for 67.1 points as the optimal threshold for differentiating CRC from adenoma among training and validation set, respectively. Sens: sensitivity; Spec: specificity; AUC: Area under the receiver operating characteristic curve. [file peerj-14-21053-s003.pdf]
