# Supplementary material for: Development and validation of machine learning-based models integrating Septin9 methylation and serum biomarkers for early detection and differentiation of colorectal cancer
Source: PeerJ. 2026 Mar 31;14:e21053. doi: 10.7717/peerj.21053 (PMC13048225; doi:10.7717/peerj.21053)
Supplement: Supplemental Information 4 [file peerj-14-21053-s004.docx]

**Supplemental Table 1. Diagnostic performance of Septin9 across different TNM Stages of CRC patients**

| **TNM Stage** | **Cases/Controls** | **AUC (95% CI)** | **Sensitivity** | **Specificity** | **PPV** | **NPV** | **LR+** | **LR-** | **MCC** |
| --- | --- | --- | --- | --- | --- | --- | --- | --- | --- |
| **Stage I** | 31 / 677 | 0.638 (0.557–0.720) | 0.290 | 0.985 | 0.474 | 0.968 | 19.65 | 0.720 | 0.349 |
| **Stage II** | 320 / 677 | 0.663 (0.636–0.689) | 0.338 | 0.985 | 0.915 | 0.759 | 22.85 | 0.672 | 0.466 |
| **Stage III** | 222 / 677 | 0.702 (0.669–0.735) | 0.414 | 0.990 | 0.929 | 0.838 | 40.08 | 0.592 | 0.557 |
| **Stage IV** | 88 / 677 | 0.844 (0.795–0.893) | 0.693 | 0.990 | 0.897 | 0.961 | 67.04 | 0.310 | 0.766 |

*Notes:* All performance metrics were calculated using the pre-defined optimal threshold for Septin9. AUC: Area under the receiver operating characteristic curve; CI: Confidence interval; PPV: Positive predictive value; NPV: Negative predictive value; LR+: Positive likelihood ratio; LR-: Negative likelihood ratio; MCC: Matthews correlation coefficient.
