# Supplementary material for: Development and validation of machine learning-based models integrating Septin9 methylation and serum biomarkers for early detection and differentiation of colorectal cancer
Source: PeerJ. 2026 Mar 31;14:e21053. doi: 10.7717/peerj.21053 (PMC13048225; doi:10.7717/peerj.21053)
Supplement: Supplemental Information 5 [file peerj-14-21053-s005.docx]

**Supplemental Table 2.** **Comprehensive diagnostic performance of the nomogram and baseline markers in the two models**

| **Models** | **Cohort** | **Analysis** | **AUC (95% CI)** | **TP / FN** | **TN / FP** | **Sens** | **Spec** | **PPV** | **NPV** | **LR+** | **LR-** | **MCC** |
| --- | --- | --- | --- | --- | --- | --- | --- | --- | --- | --- | --- | --- |
| **Model for predicting high-risk colorectal diseases** | **Training**  **(n=1200)** | Full Nomogram | 0.879 (0.860-0.898) | 533 / 192 | 426 / 49 | 0.735 | 0.897 | 0.916 | 0.689 | 7.13 | 0.30 | 0.618 |
|  |  | Age + Gender | 0.760 (0.732-0.787) | 453 / 272 | 373 / 102 | 0.625 | 0.785 | 0.816 | 0.578 | 2.91 | 0.48 | 0.402 |
|  |  | Only Septin9 | 0.678 (0.660-0.696) | 267 / 458 | 468 / 7 | 0.368 | 0.985 | 0.974 | 0.505 | 24.99 | 0.64 | 0.412 |
|  | **Validation**  **(n=514)** | Full Nomogram | 0.881 (0.852-0.910) | 238 / 74 | 175 / 27 | 0.763 | 0.866 | 0.898 | 0.703 | 5.71 | 0.27 | 0.615 |
|  |  | Age + Gender | 0.795 (0.756-0.834) | 227 / 85 | 149 / 53 | 0.728 | 0.738 | 0.811 | 0.637 | 2.77 | 0.37 | 0.456 |
|  |  | Only Septin9 | 0.685 (0.657-0.713) | 118 / 194 | 200 / 2 | 0.378 | 0.990 | 0.983 | 0.508 | 38.20 | 0.63 | 0.425 |
|  | **Training**  **(n=726)** | Full Nomogram | 0.845 (0.817–0.874) | 361 / 112 | 205 / 48 | 0.763 | 0.810 | 0.883 | 0.647 | 4.02 | 0.29 | 0.551 |
|  |  | Age + Gender | 0.674 (0.634–0.714) | 290 / 183 | 176 / 77 | 0.613 | 0.696 | 0.790 | 0.490 | 2.01 | 0.56 | 0.294 |
| **Model for differentiating CRC from adenoma** |  | Only Septin9 | 0.590 (0.555–0.625) | 140 / 333 | 238 / 15 | 0.296 | 0.941 | 0.903 | 0.417 | 4.99 | 0.75 | 0.275 |
|  | **Validation**  **(n=311)** | Full Nomogram | 0.836 (0.790–0.881) | 148 / 40 | 99 / 24 | 0.787 | 0.805 | 0.860 | 0.712 | 4.03 | 0.26 | 0.582 |
|  |  | Age + Gender | 0.648 (0.585–0.711) | 126 / 62 | 74 / 49 | 0.670 | 0.602 | 0.720 | 0.544 | 1.68 | 0.55 | 0.268 |
|  |  | Only Septin9 | 0.582 (0.530–0.634) | 56 / 132 | 115 / 8 | 0.298 | 0.935 | 0.875 | 0.466 | 4.58 | 0.75 | 0.282 |

*Notes:* AUC, area under the receiver operating characteristic curve; CI, confidence interval; TP: true positive; FN: false negatives; TN: true negative; FP: false positive; Sens: sensitivity; Spec: specificity; PPV: positive predictive value; NPV: negative predictive value; LR+: positive likelihood ratio; LR-: negative likelihood ratio; MCC: Matthews correlation coefficient. All diagnostic metrics were calculated based on the optimal cutoff value determined by the Youden Index. In both models, CRC was defined as the positive class. Normal colonoscopy controls, and adenoma was defined as the negative class in the two models, respectively.
