# Supplementary material for: Development and validation of machine learning-based models integrating Septin9 methylation and serum biomarkers for early detection and differentiation of colorectal cancer
Source: PeerJ. 2026 Mar 31;14:e21053. doi: 10.7717/peerj.21053 (PMC13048225; doi:10.7717/peerj.21053)
Supplement: Supplemental Information 6 [file peerj-14-21053-s006.docx]

**Supplemental Table 3. Multivariate logistic regression models and diagnostic performance based on binary Septin9 status**

| **Category** | **Variables / Metrics** | **Predictive Model** | **Differentiation Model** |
| --- | --- | --- | --- |
| **Predictors** |  | **OR (95% CI)** | **OR (95% CI)** |
|  | Septin9.GROUP | 25.10 (11.35 – 55.49) | *(Not Included)* |
|  | log_10_CEA | 4.36 (2.65 – 7.17) | 9.66 (5.44 – 17.16) |
|  | age | 1.06 (1.04 – 1.07) | 1.02 (1.01 – 1.04) |
|  | Hb | 0.95 (0.94 – 0.97) | 0.96 (0.95 – 0.97) |
|  | CRP | 1.07 (1.02 – 1.13) | 1.05 (1.01 – 1.09) |
|  | gender (Male) | 2.44 (1.65 – 3.61) | *(Not Included)* |
| **Model Performance** |  | **AUC (95% CI)** | **AUC (95% CI)** |
|  | Training cohort | 0.879 (0.860 – 0.898) | 0.843 (0.814 – 0.872) |
|  | Validation cohort | 0.880 (0.851 – 0.909) | 0.834 (0.789 – 0.880) |

*Note*: Odds ratios (ORs) are presented with 95% confidence intervals (CIs). AUC: Area under the receiver operating characteristic curve.
